# A Kinetic Approach of DPPH Free Radical Assay of Ferulate-Based Protic Ionic Liquids (PILs)

**DOI:** 10.3390/molecules23123201

**Published:** 2018-12-05

**Authors:** Nur Afiqah Ahmad, Khairulazhar Jumbri, Anita Ramli, Noraini Abd Ghani, Haslina Ahmad, Jun Wei Lim

**Affiliations:** 1 Department of Fundamental and Applied Sciences, Universiti Teknologi PETRONAS, Seri Iskandar 32610, Perak, Malaysia; nurafiqahahmad306@gmail.com (N.A.A.); anita_ramli@utp.edu.my (A.R.); noraini.ghani@utp.edu.my (N.A.G.); junwei.lim@utp.edu.my (J.W.L.); 2 Centre of Research in Ionic Liquids (CORIL), Universiti Teknologi PETRONAS, Seri Iskandar 32610, Perak, Malaysia; 3 Department of Chemistry, Faculty of Science, Universiti Putra Malaysia, UPM Serdang 43400, Selangor, Malaysia; haslina_ahmad@upm.edu.my

**Keywords:** protic ionic liquids, antioxidant, DPPH radical scavenging, kinetic, antiradical efficiency

## Abstract

The antiradical efficiency (AE) and kinetic behavior of a new ferulate-based protic ionic liquids (PILs) were described using 2,2-diphenyl-1-picrylhydrazyl (DPPH) free radical assay. The reduction of the DPPH free radical (DPPH•) was investigated by measuring the decrease in absorbance at 517 nm. The time to reach steady state for the reaction of parent acid (ferulic acid) and synthesized PILs with DPPH• was continuously recorded for 1 h. Results revealed that the AE of 2-butylaminoethanol ferulate (2BAEF), 3-dimethylaminopropanol ferulate (3DMAPF) and 3-diethylaminopropanol ferulate (3DEAPF) PILs have improved compared to ferulic acid (FA) as the reaction class changes from low to medium. This attributed to the strong hydrogen abstraction occurred in the PILs. Furthermore, these PILs were found to have a good kinetic behavior compared to FA due to the high rate constant (*k*_2_) (164.17, 242.84 and 244.73 M^−1^ s^−1^, respectively). The alkyl chain length and more alkyl substituents on the nitrogen atom of cation were believed to reduce the cation-anion interaction and speed up the hydrogen atom transfer (HAT) and electron transfer (ET) mechanisms; hence, increased rate constant was observed leading to a strong antioxidant activity of the synthesized PILs.

## 1. Introduction

Phenolic compounds are secondary metabolites that exist in plant tissue. Typically, these compounds act as an antioxidant in a biological system as their ability is to reduce oxidative-stress resulting in chronic disease [1]. The two major classes of phenolic compounds are flavonoid and non-flavonoids [2]. Phenolic acids are the most natural non-flavonoid antioxidants highly found in plants. They had been widely used in health, cosmetics, food and pharmaceutical industries [3,4,5,6] due to their competent biological properties such as antioxidant, anti-cancer, anti-carcinogenic or anti-mutagenic, anti-bacterial, anti-viral and anti-inflammatory [7,8,9,10,11]. Phenolic acids were derived from two major derivatives known as benzoic and cinnamic acid. Both derivatives were distinguished based on the structure of their compounds [12].

Ferulic acid (FA) is a chemical compound derived from a cinnamic acid derivative. This compound is usually found in herbal medicines and plants [13]. Besides, FA is a phenolic acid with low toxicity since it is easy to be absorbed and metabolized by the human body [14]. Moreover, this compound is commonly used in food, pharmaceutical and cosmetic products due to its strong antioxidant properties [15,16]. Furthermore, it has been reported to have other several biological properties such as anti-inflammatory, antimicrobial, anticancer, anti-diabetes and anti-neurodegenerative [17,18,19]. On top of that, this compound is very reactive towards reactive oxygen species (ROS), lipid oxidation and 2,2-diphenyl-1-picrylhydrazyl (DPPH) free radical [20].

From the previous study, several methods have been applied for assessing the antioxidant activity of a compound. For instance, oxygen radical absorbance capacity (ORAC), total radical antioxidant parameter (TRAP), ferric reducing antioxidant power (FRAP), Cupric reducing antioxidant power (CUPRAC), lipid peroxidation, 2,2′-azinobis(3-ethylbenzthiazoline-6-sulfonic acid (ABTS) and 2,2-diphenyl-1-picrylhydrazyl (DPPH) radical scavenging assay [21,22,23,24].Nevertheless, the DPPH radical scavenging assay is the most common method used due to its stable, easy to perform and commercial availability compared to other methods [25]. This assay was based on the principle of reduction of DPPH free radical by accepting a hydrogen atom from the scavenger compound; hence, the color was seen changing from violet to yellow [26,27]. The DPPH free radical is classified as a naturally stable nitrogen radical that might have a different reaction kinetic with antioxidant compounds [28].

The kinetic reaction of the antioxidant compound is really important as the free radical has a short half-life [29]. Indeed, most studies have focused on the kinetic behavior of compounds to determine their effective antioxidants [30,31,32]. Recently, modifications on phenolic acid in the form of ionic liquids (ILs) have increased its popularity. This is because ILs were known as a tuneable compound where their properties can be tuned according to the types of cation and anion used [33]. Czerniak et al. synthesized gallate-based ILs to enhance the performance of the antioxidant. From their finding, the antioxidant activity of the synthesized ILs were seen affected by the intermolecular hydrogen bond that present due to the linear chain and stearic effect of the structure [34]. The same research group has then studied the dicationic based ILs and found that as the number of the active group increases on the structure, the antioxidant activity of the synthesized compound increased [35]. Furthermore, there is another study reporting the abietate-based ILs. However, the antioxidant activity was only focused on the measurement of the EC_50_ of the synthesized compound [36]. Therefore, in this work, five new ferulate-based PILs were synthesized to enhance the performance of antioxidant. Apart from calculating the EC_50_, antiradical efficiency (AE) and rate constant of the synthesized PILs were also discussed to determine the effective PILs toward DPPH free radical (DPPH•).

## 2. Results and Discussion

The antioxidant activity of the synthesized PILs, which are 2-methylaminoethanol ferulate (2MAEF), 2-propylaminoethanol ferulate (2PAEF), 2-butylaminoethanol ferulate (2BAEF), 3-dimethylaminopropanol ferulate (3DMAPF) and 3-diethylaminoethanol ferulate (3DEAPF) was determined by calculating the EC_50_, TEC_50_, antiradical efficiency (AE) and rate *constant* (*k*_2_).

### 2.1. EC_50_

**Figure 1:**
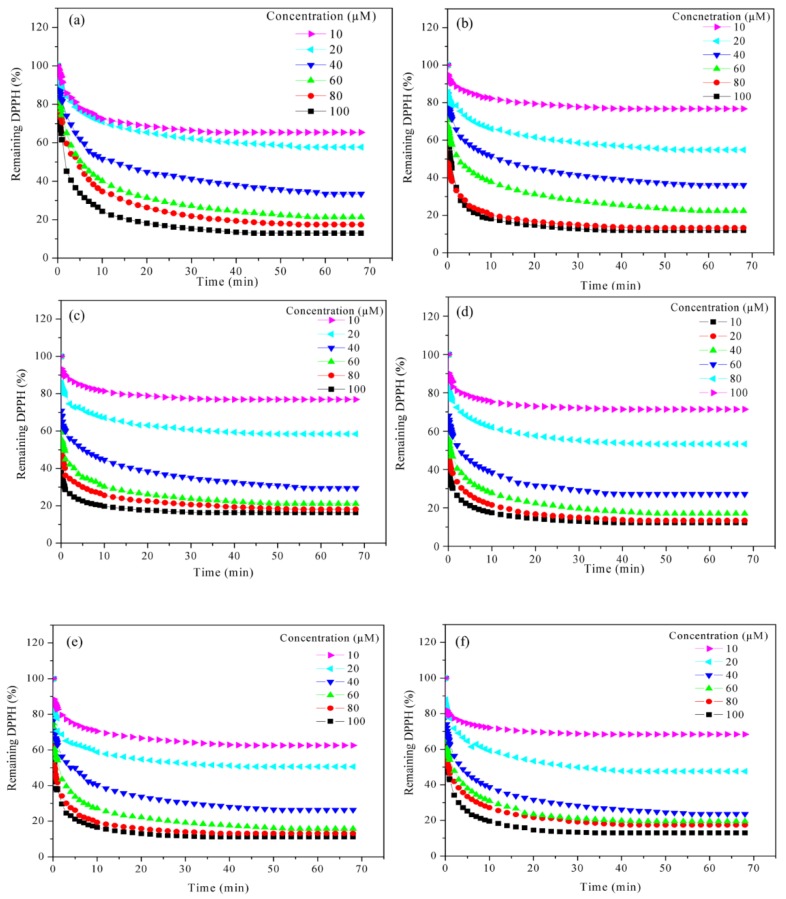
Reaction kinetic between 60 µM of DPPH• and different concentration FA and synthesized PILs: (**a**) FA; (**b**) 2MAEF; (**c**) 2PAEF; (**d**) 2BAEF; (**e**) 3DMAPF; (**f**) 3DEAPF.

EC_50_ is the maximum concentration needed to reduce half of the DPPH free radical (DPPH•) at steady state, which is known as half-maximal effective concentration. Figure 1a–1f depict the reaction between 60 µM DPPH• with different concentrations of FA and PILs. The percentage of remaining DPPH• was rapidly decreased as the concentration of FA and PILs increases. Increase in concentration will also increase the total number of molecules. Hence, the scavenging activity of the active compound was increased due to the presence of more active groups in the reaction medium [37].

The effective antioxidant activity of the synthesized PILs and FA was determined by measuring the EC_50_. The EC_50_ was calculated to express the scavenging ability of 50% DPPH• at steady state (Figures S1–S6). The compound with lower EC_50_ will exhibit higher and good antioxidant activity. The summarized results in Table 1 demonstrate that the synthesized PILs being a more potent antioxidant as the EC_50_ was ranging from 18.40 ± 0.12 µM to 25.00 ± 0.20 µM, which is lower than FA (26.00 ± 0.10 µM). This was believed to occur due to the presence of the extra active groups (-OH and -NH) on the cation and anion structure of PILs [38]. The active group will help to neutralize the radical on the nitrogen atom of the DPPH• by transferring of hydrogen atom via hydrogen abstraction process [39].

The EC_50_ of the synthesized five ferulate-based PILs was seen only slightly different since all of them have the same number of active groups attached on the ion-pair structure. However, the order of the reactivity was increased from 2MAEF < 2PAEF < 2BAEF < 3DMPAF < 3DEAPF with the EC_50_ of 25.00 ± 0.20, 24.30 ± 0.14, 22.00 ± 0.17, 20.40 ± 0.05 and 18.40 ± 0.12 µM, respectively. The low presence of DPPH• at steady state was expected to affect the reactivity since the percentage remaining DPPH• at steady state also depends on the ability of the compound to transfer the hydrogen atom in a fast manner, thus leading to the decrease of concentration needed to scavenge 50% of DPPH• (EC_50_). This is further discussed in the next section (TEC_50_).

### 2.2. TEC_50_

The time to reach the steady state (TEC_50_) was considered to determine the antioxidant effect of the tested compound. The faster the reaction reaches a steady state, the more efficient is the antioxidant. The calculation of TEC_50_ shows in Figures S7–S12. According to a previous study, the FA was classified as a medium reaction since the time needed to reach a steady state is around 30 min to 1 h [41]. From Table 1, the TEC_50_ of FA (56 min) did not differ greatly with the synthesized PILs (44–53 min), whereas the class of reaction remained unchanged.

Moreover, the selection of quaternary-ammonium cation had caused a slow diffusion of PILs due to the narrow-like shape or bulky structure in the substitution of an alkyl group at the nitrogen atom. Tokuda et al. examined the diffusion coefficient between the aromatic-cation based and quaternary-ammonium cation based ILs [42]. From their findings, the aromatic-cation based ILs had shown a larger diffusion coefficient (15.0 × 10^−7^ cm^2^ s^−1^) than the quaternary-ammonium cation based (9.3 × 10^−7^ cm^2^ s^–1^) as the presence of aromatic ring led to the formation of broad-like shape and fast diffusion of ion to occur easily [43]. Since the transfer of hydrogen atom is diffusion controlled reaction, the slow diffusion of quaternary-ammonium cation had affected the TEC_50_ of synthesized PILs [44].

**Figure 2:**
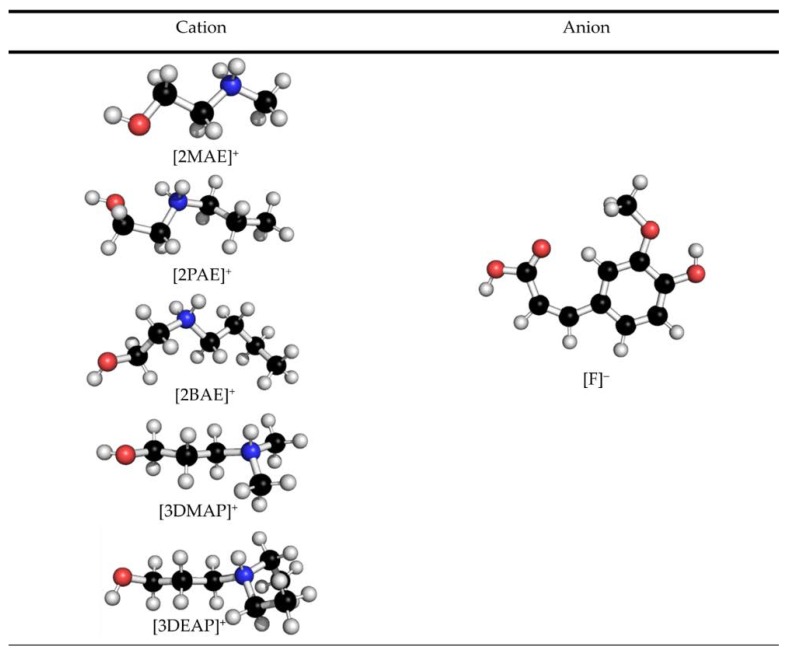
Cation and anion structure of the synthesized PILs.

However, in the case of cation structure, [2BAE]^+^, [3DMAP]^+^ and [3DEAP]^+^ need lesser time to react with the DPPH• than [2MAE]^+^ and [2PAE]^+^ (45, 44, 42, 50, and 53 min, respectively). The increase in alkyl chain length and more alkyl substituent on the nitrogen atom of the cation structure caused the formation of a more flexible structure that can reduce the strength of intermolecular hydrogen bond between the ion pairs and the effect of steric hindrance, thus allowing a fast transfer of hydrogen atom and increasing the scavenging activity of DPPH•. Furthermore, both [2MAE]^+^ and [2PAE]^+^ have a small and rigid structure with high tendency to form a strong hydrogen bond with the solvent (methanol) and ferulate anion [F]^−^, which led to a slow release of hydrogen atom of -NH group [44]. The cation and anion structure of synthesized PILs shows in Figure 2.

### 2.3. Antiradical Efficiency (AE)

The antiradical efficiency (AE) is the significant parameter measured to determine the effectiveness of antioxidant activity. This parameter is a kinetic-based study as the measurement comprised the potency (EC_50_) and the reaction time (TEC_50_). The AE was calculated using the formula in the Equation (1)

(1) $$AE=\frac{1}{EC_{50}\times TEC_{50}}$$

Based on the formula given, the lower EC_50_ and TEC_50_ will give a higher AE. Table 1 shows that the AE of synthesized PILs was ranged from 0.75 ± 0.15 to 1.29 ± 0.07 µM, which is higher than the FA (0.70 ± 0.05µM). A higher AE indicates a more efficient antioxidant due to the higher reactivity of active group with the DPPH•. This is expected to occur because of the presence of the extra active group and fast reaction. According to the AE classification (Table 2) [45], the AE of the compound followed the order of 3DEAPF ≥ 3DMAPF ≥ 2BAEF > 2PAEF ≥ 2MAEF > FA. The class of the AE was slightly enhanced from low to medium class as the alkyl chain length and substituent of the cation structure increase. The short alkyl chain length and more hydrogen substituents on the nitrogen atom of cation formed a small structure of PILs that can enhance the presence of the PILs molecules in the reaction system. Hence, promote to random molecular packing that reduced the accessibility to DPPH• due to the crowding effect of PILs molecules around the DPPH• molecules [46]. Moreover, the changes in the classes may be reflected by the fast rate of reaction, which is very important in biological system since the free radical only has a short lifetime [29].

### 2.4. Stoichiometry of Reaction 

The stoichiometric is the amount of antioxidant needed to reduce 100% of DPPH• in which the value was calculated by multiplying the EC_50_ (in molar ratio) with 2 and the inverse values indicating the moles of DPPH• were reduced by 1 mol of antioxidant [41,47]. This parameter is also known as a stoichiometric factor (*n*) and was calculated to determine the kinetic behavior of the compound. The *n* of FA (Table 3) was seen in agreement with the value obtained by Brand-Williams et al. [47]. As predicted, the *n* value of PILs was higher than FA since the value was calculated from the EC_50_. However, the number of active group present on the structure was negligible as the *n* value was affected by the structure reactivity. This has been proven by the findings of Xie et al. in their study on the correlation of initial rate with stoichiometry [46].

### 2.5. Kinetic Behaviour of PILs Towards DPPH•

Fast initiate step of synthesized PILs and FA towards DPPH• was determined to study the effectiveness of the antioxidant. Generally, the DPPH• reacts with the antioxidant compound (ArOH) via two different reaction mechanisms. The first mechanism is called hydrogen transfer (HAT), which is a direct abstraction of hydrogen atom reaction. The second mechanism is the electron transfer reaction (ET) from ArOH or its anion (ArO^−^) to DPPH• which also known as electron abstraction [27]. The scavenging activity is represented by the equation as follows:

(1) HAT mechanism

(2) ArOH+DPPH•→ArO•+H−DPPH

(3) ArO•+DPPH•→product

This mechanism neutralized the DPPH• by directly reacting with the antioxidant compound to form radical of phenolic antioxidant. In the reaction the bond dissociation energy (BDE) had affected HAT mechanism.

(2) ET mechanism

(4) $$ArOH+X•\to ArOH•{}^{+}+X^{-}$$

(5) $$ArOH•{}^{+}\to ArO•+H^{+}$$

The ET mechanism has two-step reaction in which the first step involved the formation of phenolic cationic radical and an anionic compound. Then, in the second step, the antioxidant was decomposed into phenolic radical and proton [27]. This mechanism depends on the ionization potential (IP).

The kinetic reaction of scavenging DPPH• is depend on the relative concentration of reactants [41]. In the present study the concentration of DPPH• and PILs was considered. Since the concentration of DPPH• was fixed at 60 µM, the reaction followed the pseudo-first order as the increasing concentration of PILs and FA in the reaction medium was in a large excess. The reaction was defined as

(6) $$-\frac{d\left[\mathrm{DPPH}•\right]}{dt}=k_{1}\left[\mathrm{DPPH}•\right]$$

**Figure 3:**
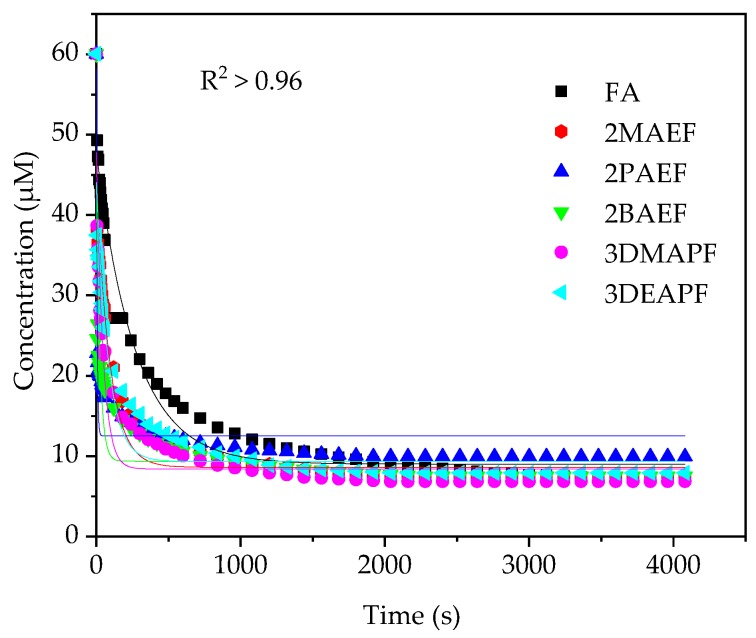
Curve fitting of pseudo first order rate constant (*k*_1_) of synthesized PILs and FA.

*k*_1_ is the rate constant obtained from the slope of non-linear pseudo first order (Figure 3). The equation was written as:

(7) $$A=A_{0}e^{-k_{1}t}$$

where *A* is the DPPH• concentration, *t* is time and *A*_0_ represents DPPH• concentration at *t* = 0. The *k*_1_ is rate constant.

Therefore, the rate of reaction was respected to the concentration of DPPH•.

However, the *k*_1_ value was also depended on concentration of PILs. Thus, the rate of DPPH• scavenging could be defined as

(8) $$-\frac{d\left[\mathrm{DPPH}•\right]}{dt}=k_{1}\left[\mathrm{DPPH}•\right]=nk_{2}\left[\mathrm{DPPH}•\right]\left[ArOH\right]$$

*n* correspond to the stoichiometric factor of ArOH and *k*_2_ is the rate constant. By considering the stoichiometric factor (*n*), *k*_2_ value was calculated from the equation written as

(9) $$k_{2}=\frac{k_{1}}{n\;\left[ArOH\right]}$$

Table 4 shows that the 2BAEF, 3DMAPF and 3DEAPF were found to have a higher rate constant (*k*_2_) (164.17, 242.84 and 244.73 M^−1^ s^−1^, respectively) than FA (109.48 M^−1^ s^−1^). However, another two PILs (2MAEF and 2PAEF) has shown lower reaction towards DPPH• as they have lower *k*_2_ (86.89 and 107.20 M^−1^ s^−1^). The structure-activity relationship (SAR) was suggested to affect the *k*_2_ of the synthesized PILs and FA because the different structure will react differently to scavenge the DPPH• [48]. Furthermore, in this study, the different cation structure was predicted to influence the rate constant of PILs. As previously discussed, the rate constant was controlled by the HAT and ET mechanisms. Klein et al. reported that the mechanism of the antioxidant amine type structure (-NH) relies upon the N-H bond dissociation energy (BDE) and ionization potential (IP) [49]. They concluded that the BDE and IP of N-H bond are highly dependent on substituent group. Lalevée et al. 2002 reported the trend of the BDE for amine group. From their finding, the strength of the BDE decreased as the bulkiness and *N*-alkylation of the amine group increases. This indicates the formation of stable aminyl radical from σC–C bond by hyperconjugation reaction [50]. For instance, 3DMAPF and 3DEAPF were found to have strong reactivity compared to 2MAEF, 2PAEF and 2BAEF. This corresponds to the alkyl substituent on the nitrogen atom that reduced the BDE and IP as weak intramolecular hydrogen bond occurred between the nitrogen and hydrogen atom of -NH group. Besides, the lower rate constant of 2MAEF and 2PAEF was observed due to the strong intermolecular interaction between the ion pairs leading to compact structure, thus hindering the radical scavenging of the -OH and -NH group on PILs [51]. The calculated *k*_2_ of FA were in agreement with previous work [52]

## 3. Materials and Methods

### 3.1. Materials

Ferulic acid (FA, purity 98%) and 3-diethylamino-1-propanol (3DEAP, purity 98%) were purchased from Across (Shah Alam, Malaysia). 2,2-diphenyl-1-picrylhydrazyl (DPPH, purity 95%), 2-(propylamino)ethanol (2PAE, purity, 96%) and methanol were supplied by Sigma Aldrich (Darmstadt, Germany). 2-(Methylamino)ethanol (2MAE, purity 98%), 2-(Butylamino)ethanol (2BAE, purity 98%) and 3-dimethylamino-1-propanol (3DMAP, purity 99%) were all purchased from Merck (Darmstadt, Germany). All chemicals were used as received without further purification.

### 3.2. Synthesis of PILs

The PILs were synthesized by mixing an equimolar of parent acid (FA) with five different alkanolamine bases (2MAE, 2PAE, 2BAE, 3DMAP and 3DEAP) through neutralization reaction. A 6.9 g of FA (0.05 mol, in excess) was dissolved with 50 mL hot distillate water in 100 mL two necks round bottom flask equipped with a reflux condenser and the solution was stirred vigorously. Excess of FA was used to make sure the neutralization reaction occurs completely. Then, an equimolar of the base was slowly introduced into to the FA solution. The mixture was then stirred for 24 h and the unreacted acid was removed by filtration. The water was removed by using rotatory evaporator at 50 °C (60 mbar) for 5 h. Then, the synthesized PILs were further dried under vacuum for overnight. All PILs were successfully characterized by using NMR (Selangor, Malaysia), FTIR (Kyoto, Japan), LC-MS (Bayan Lepas, Penang, Malaysia), and thermal analysis (Kyoto, Japan).

### 3.3. Preparation of Stock Solution

The five-ferulate based PILs which are 2-methylaminoethanol ferulate (2MAEF), 2-propylaminoethanol ferulate (2PAEF), 2-butylaminoethanol ferulate (2BAEF), 3-dimethylaminopropanol ferulate (3DMAPF) and 3-diethylaminoethanol ferulate (3DEAPF), were successfully synthesized through neutralization reaction method. Afterwards, 20 mM of synthesized PILs was prepared in methanol solution followed by serial dilution into six lower concentrations (100, 80, 60, 40, 20 and 10 mM) using 10 mL of volumetric flask. The final concentration of PILs after added into the DPPH solution was ranging from 10 to 100 µM. For the free radical stock solution, 2.37 mg of DPPH was dissolved in a methanol and diluted into 100 mL of volumetric flask to prepare 60 µM of DPPH solution. As a precaution step, the solution was freshly prepared and kept in a dark condition at ambient temperature during the analysis.

### 3.4. DPPH free Radical Assay

**Figure 4:**
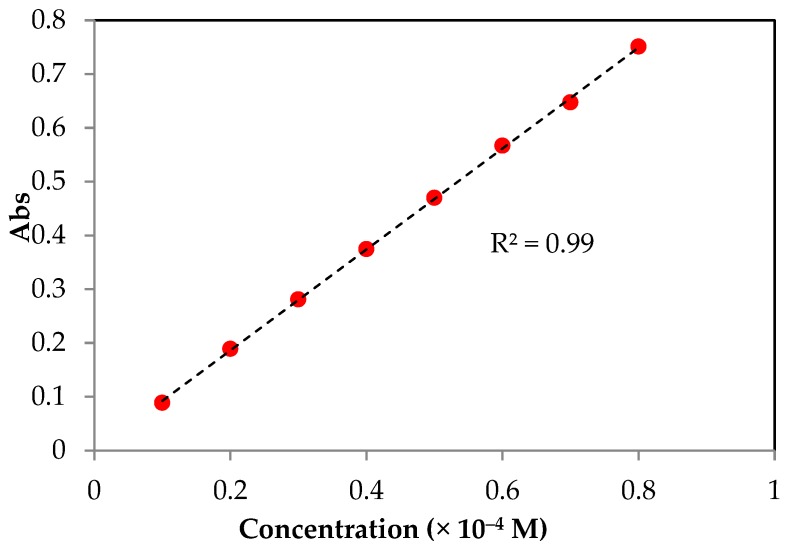
Calibration curve of DPPH solution.

The modified 2,2-diphenyl-1-picrylhydrazyl (DPPH) free radical assay has followed the method determined by Brand-Williams et al. [47]. Based on this assay, the color change of DPPH solution (in methanol) from violet to yellow was observed using Thermo Fisher UV-Vis spectrometer (Waltham, MA, USA) by measuring the change of absorbance at a wavelength 517 nm. The yellow color indicated the reduction of DPPH free radical (DPPH•). Basically, a 3000 µL of DPPH solution with a concentration of 60 µM was pipetted into a quartz cuvette with the initial absorbance measured. Afterwards, 30 µL of the synthesized PILs with varied final concentration (10–100 µM) was added and mixed rapidly. Then, the decrease in absorbance was recorded every 5 s for 10 min and continued for every 2 min until it reaches a steady state (around 1 h). The exact concentration of DPPH• in the reaction medium was determined from a calibration curve (Figure 4) with a linear regression as follows:

$$A_{517\;nm}=9385.30\;{\left[\mathrm{DPPH}•\right]}_{t}-1.5\times 10^{-3}$$

The percentage of remaining DPPH• at steady state was calculated using the formula in Equation (10):

(10) $$\%\;\mathrm{DPPH}{•}_{rem}=\frac{A_{f}}{A_{0}}\times 100$$

where DPPH•_*rem*_ is the percentage of remaining DPPH• at steady state, while *A*_0_ and *A_f_* are the initial and final absorbance of DPPH• at 517 nm.

## 4. Conclusions

In this study, the antiradical efficiency (AE) and rate constant of ferulate-based protic ionic liquids (PILs) have been successfully studied using DPPH free radical assay to determine the kinetic behavior. Based on the results obtained, the AE class has changed from low to medium for 2BAEF, 3DMAPF and 3DEAPF, which was due to the strong HAT and ET on the cation structure of PILs that helped to increase the scavenging of DPPH•. Besides, these three PILs also demonstrated a remarkably higher kinetic reaction with the *k*_2_ values of 164.17, 242.84 and 244.73 M^−1^ s^−1^, respectively. The fast HAT and ET reaction mechanisms occurred as more substituents of alky group were present on the nitrogen atom that weakens the interaction of ion-pairs; thus strong and effective of antioxidant activity of the synthesized PILs was observed. Therefore, the synthesized of PILs with a bulkier and larger cation structure could be a good potential DPPH• scavenger for antioxidant activity.

## Figures and Tables

**Table 1:** Parameters of radical scavenging activity of parent acid (FA) and synthesized PILs: EC_50_ (µM), TEC_50_ and antiradical efficiency, AE (×10^−3^)

Sample	EC_50_ (µM)	TEC_50_	Antiradical Efficiency, AE (×10^−3^)	Reference
FA	24.7 ± 0.40	53	0.80 ± 0.00	[40]
FA	26.0 ± 0.10	56	0.70 ± 0.05	This work
2MAEF	25.0 ± 0.20	53	0.75 ± 0.15	This work
2PAEF	24.3 ± 0.14	50	0.82 ± 0.05	This work
2BAEF	22.0 ± 0.17	45	1.01 ± 0.08	This work
3DMAPF	20.4 ± 0.05	44	1.11 ± 0.03	This work
3DEAPF	18.4 ± 0.12	42	1.29 ± 0.07	This work

**Table 2:** Classification of antiradical efficiency (AE) for antioxidant compound.

Classification	Value AE
Low	AE ≤ 1.0 × 10^−3^
Medium	1.0 × 10^−3^ < AE ≤ 5.0 × 10^−3^
High	5.0 × 10^−3^ < AE ≤ 10.0 × 10^−3^
Very high	AE > 10.0 × 10^−3^

**Table 3:** Stoichiometric factor of FA and PILs calculated from the EC_50_ value.

Sample	EC_50_ (µmol of Antioxidant/ µmol of DPPH)	Stoichiometric Factor (*n*) ^1^
FA	0.43 (0.42 ^2^)	1.20
2MAEF	0.41	1.22
2PAEF	0.40	1.25
2BAEF	0.36	1.39
3DMAPF	0.33	1.52
3DEAPF	0.30	1.67

^1^ Calculated as *n* = 1/(EC_50_ × 2), ^2^ Data from reference [47].

**Table 4:** Rate constant, *k*_2_ (M^−1^ s^−1^) and stoichiometric factor (*n*) for reaction medium of DPPH• (60 µM) and 100 µM of FA or synthesized PIL.

Sample	Stoichiometric Factor (*n*)	*k*_2_ (M^−1^ s^−1^)
FA	1.16	109.48
2MAEF	1.22	86.89
2PAEF	1.25	107.20
2BAEF	1.39	164.17
3DMAPF	1.51	242.84
3DEAPF	1.67	244.73

## Supplementary Materials

Figures S1–S6: Extrapolation graph for EC_50_ of FA and synthesized PILs, Figures S7–S12: Extrapolation graph for TEC_50_ of FA and synthesized PILs.
