# Supplementary material for: A Kinetic Approach of DPPH Free Radical Assay of Ferulate-Based Protic Ionic Liquids (PILs)
Source: Molecules. 2018 Dec 5;23(12):3201. doi: 10.3390/molecules23123201 (PMC6321392; doi:10.3390/molecules23123201)

Figure S1

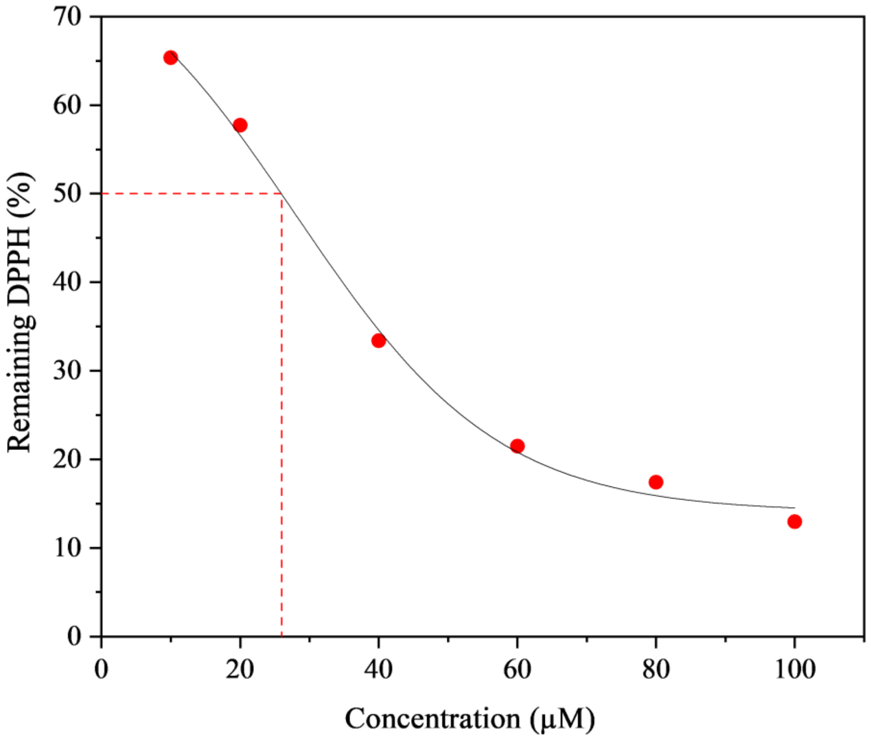

1

Figure S2

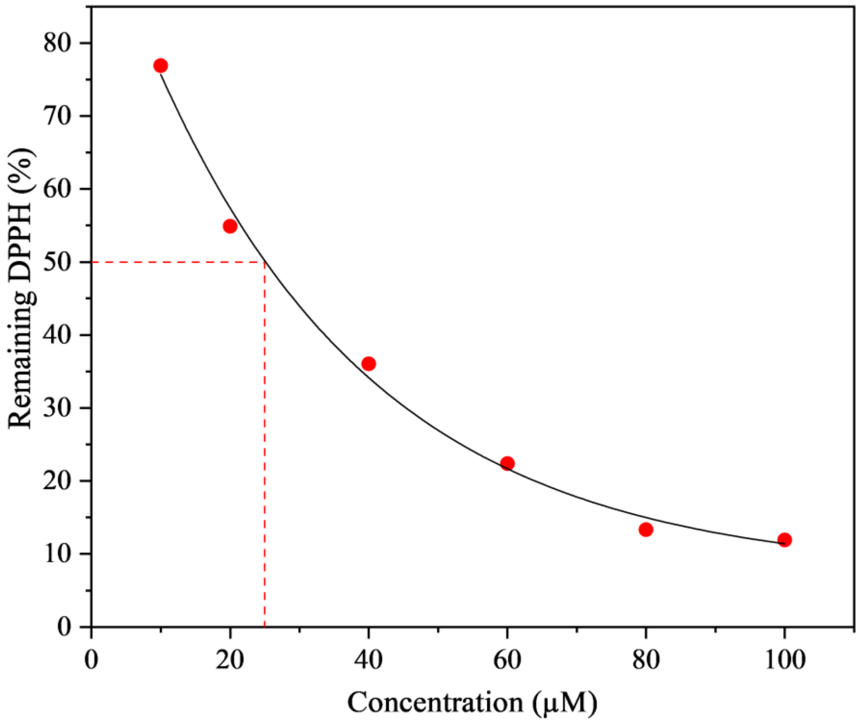

2

Figure S3

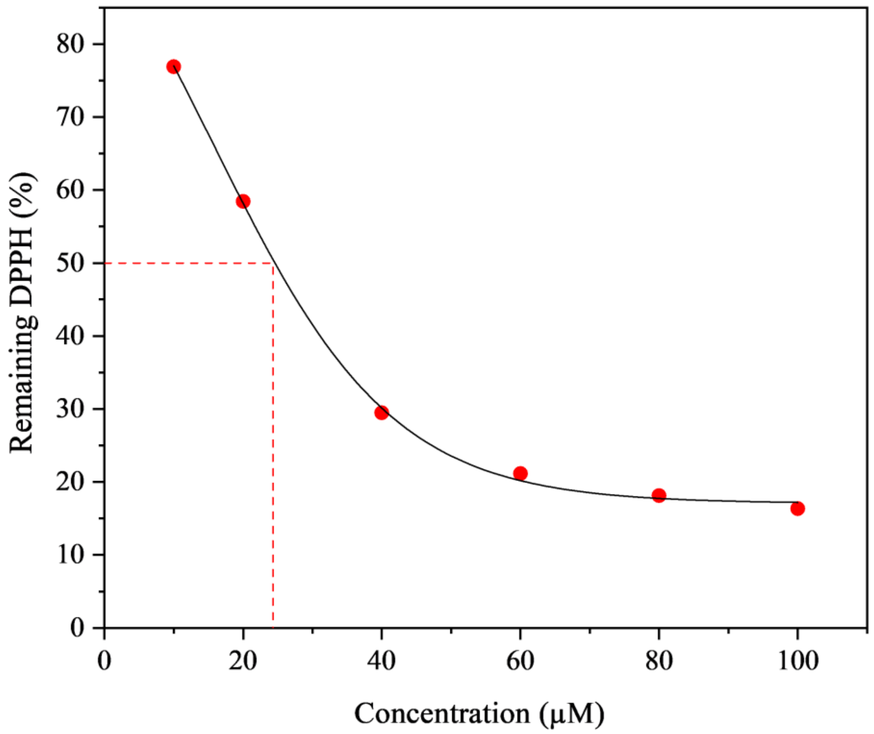

Figure S4

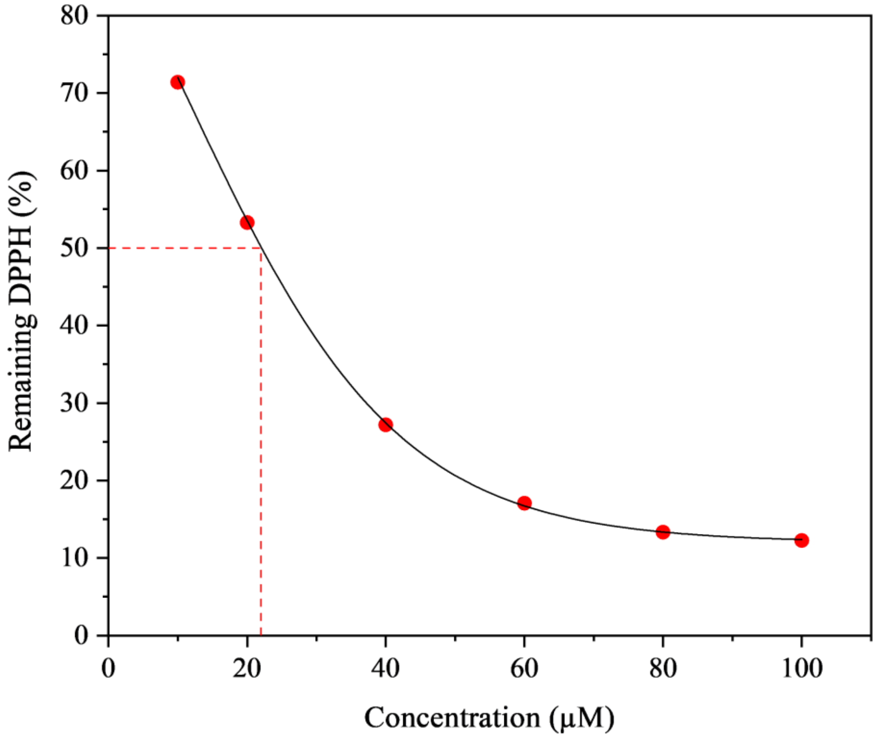

Figure S5

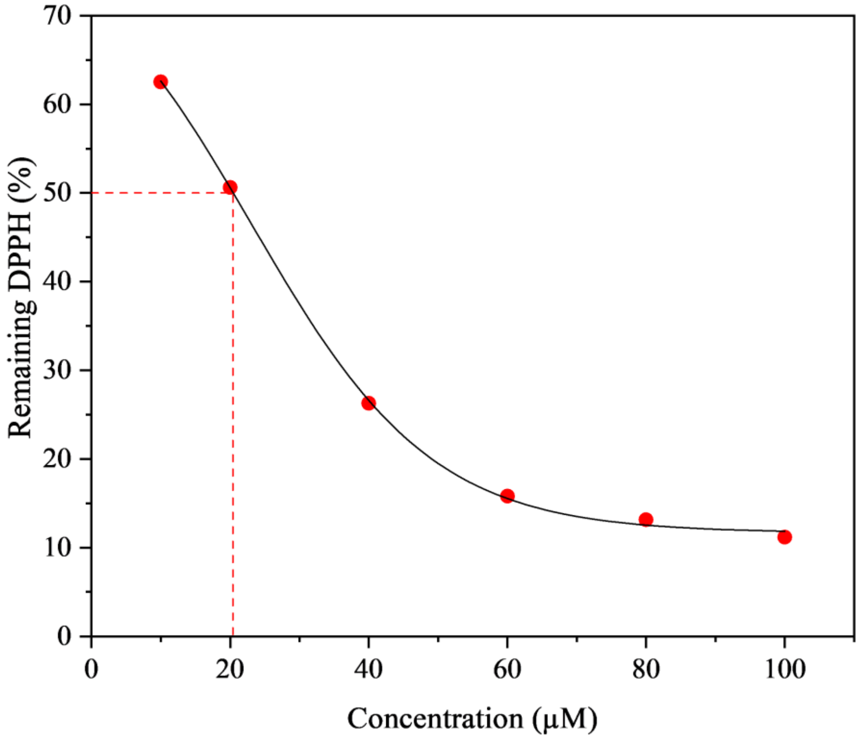

2

Figure S6

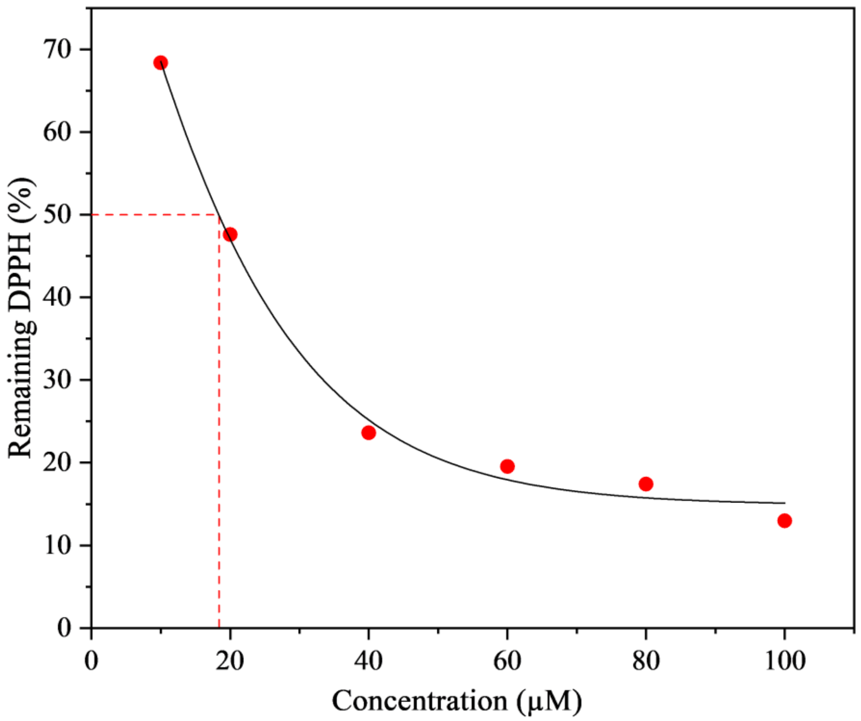

2

Figure S7

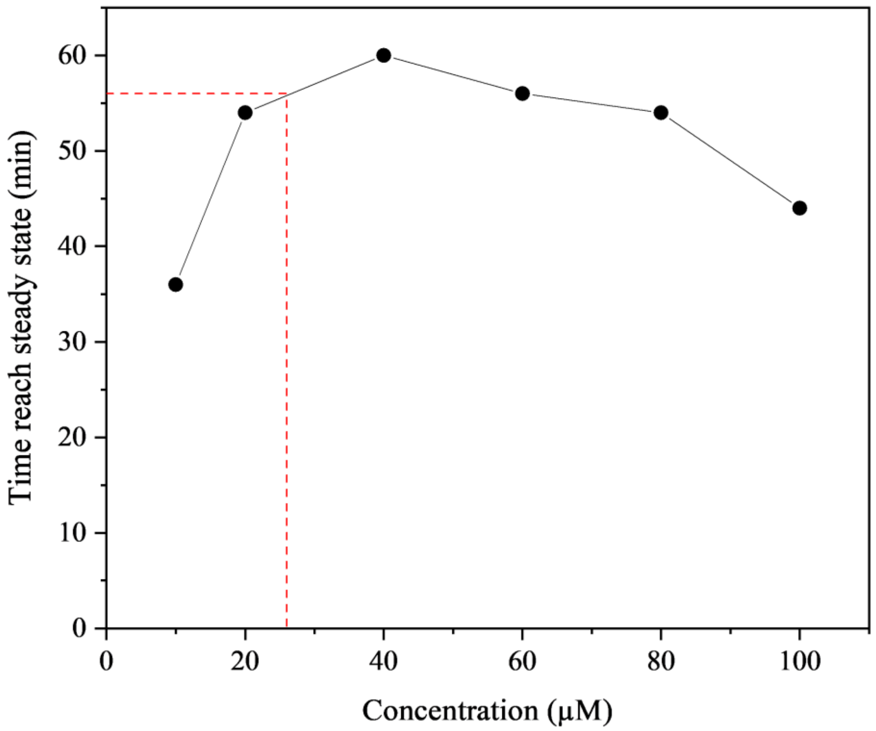

2

Figure S8

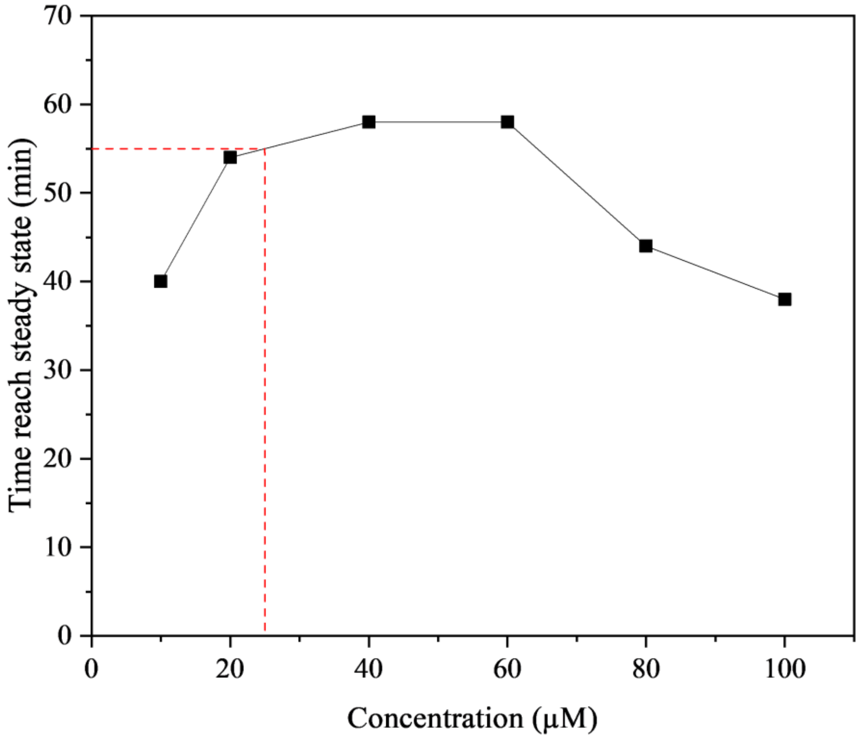

1

Figure S9

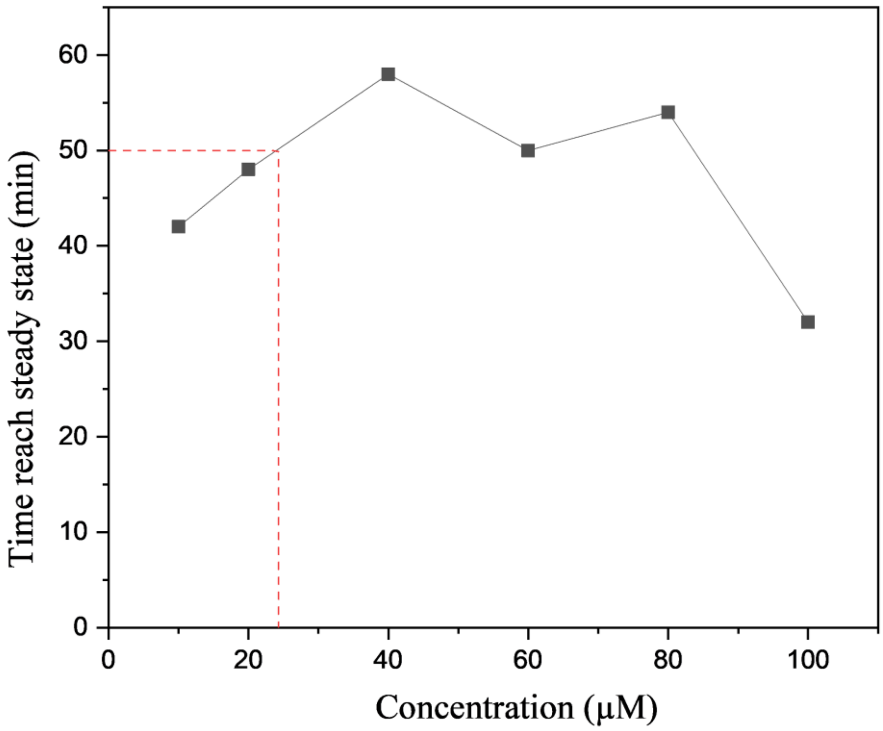

3

Figure S10

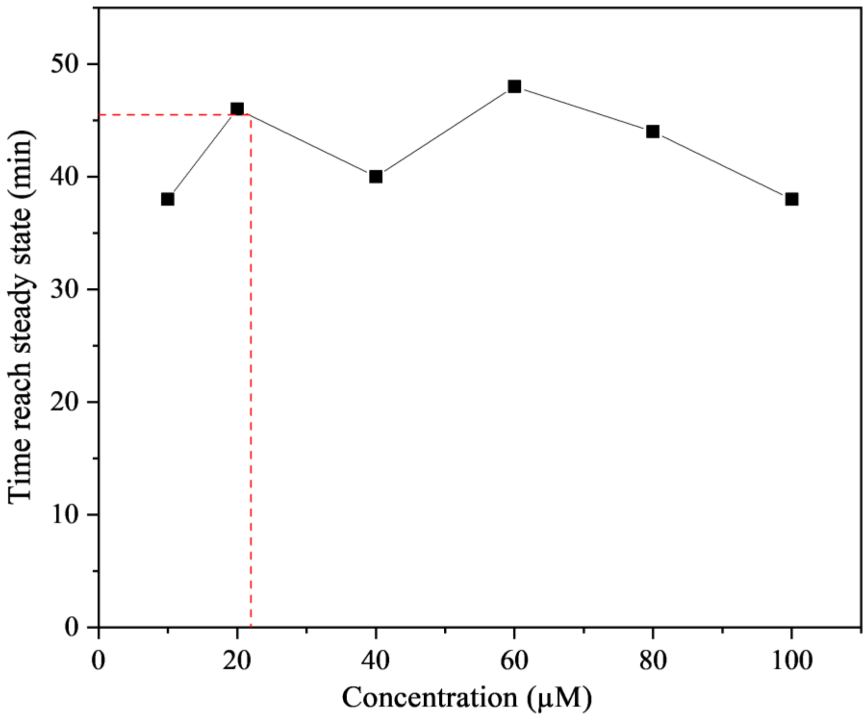

1

Figure S11

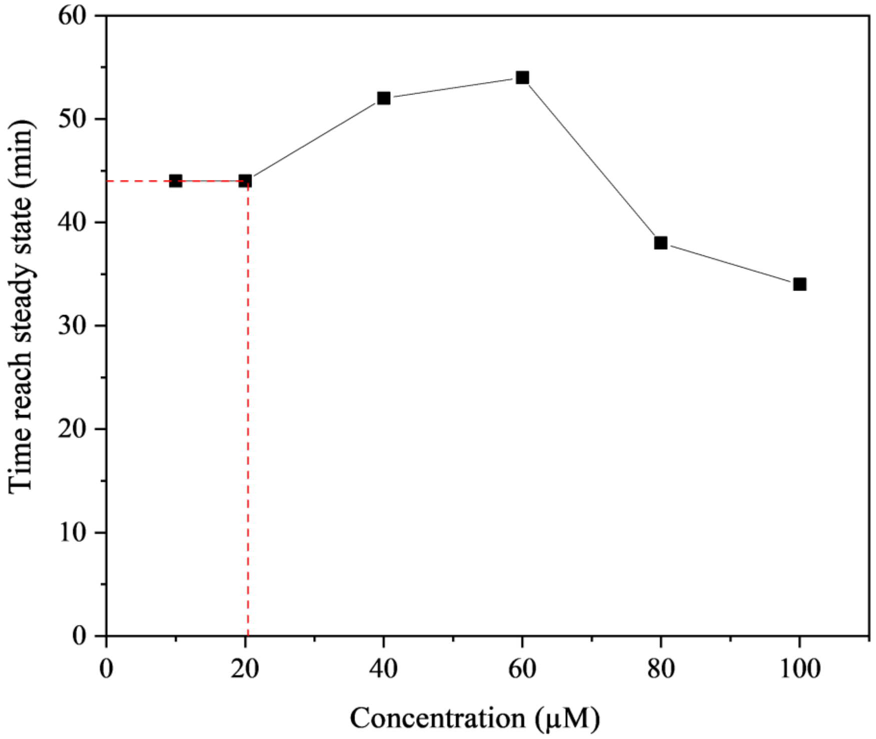

Figure S12

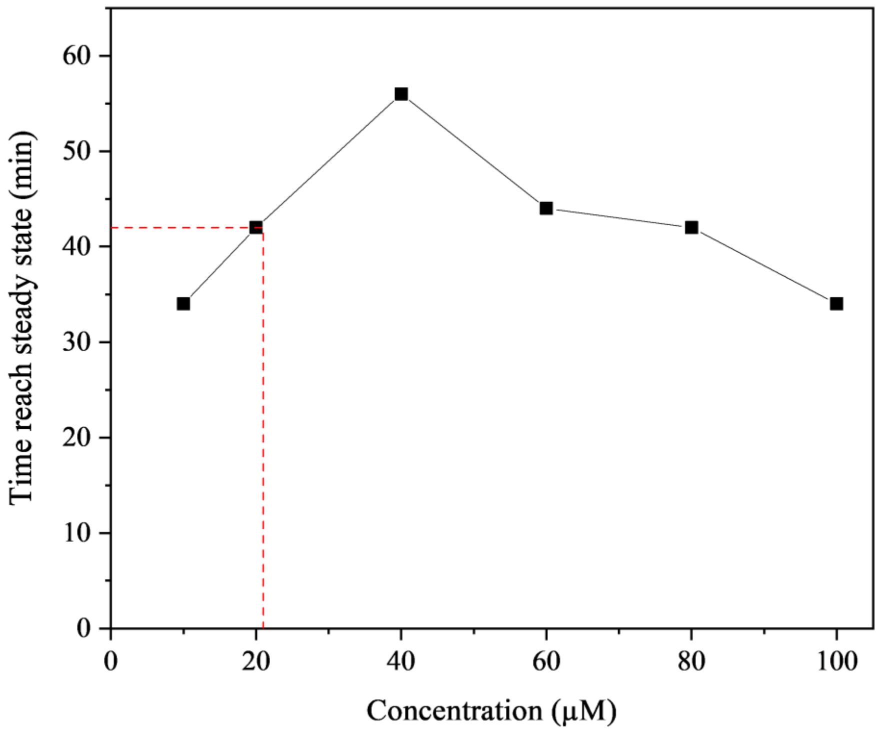

Supplement: Supplementary file 1 [file molecules-23-03201-s001.pdf]
